# The Role of Mitochondrial Dynamics and Mitophagy in Carcinogenesis, Metastasis and Therapy

**DOI:** 10.3389/fcell.2020.00413

**Published:** 2020-06-10

**Authors:** Yigang Wang, Hui-Hui Liu, Yu-Ting Cao, Lei-Lei Zhang, Fang Huang, Cong Yi

**Affiliations:** ^1^Xinyuan Institute of Medicine and Biotechnology, School of Life Sciences and Medicine, Zhejiang Sci-Tech University, Hangzhou, China; ^2^Department of Pathology, Zhejiang Provincial People’s Hospital, People’s Hospital of Hangzhou Medical College, Hangzhou, China; ^3^Department of Biochemistry, Hepatobiliary and Pancreatic Surgery, The First Affiliated Hospital, Zhejiang University School of Medicine, Hangzhou, China

**Keywords:** mitochondria, mitochondrial dynamics, mitophagy, carcinogenesis, therapy

## Abstract

Mitochondria are key cellular organelles and play vital roles in energy metabolism, apoptosis regulation and cellular homeostasis. Mitochondrial dynamics refers to the varying balance between mitochondrial fission and mitochondrial fusion that plays an important part in maintaining mitochondrial homeostasis and quality. Mitochondrial malfunction is involved in aging, metabolic disease, neurodegenerative disorders, and cancers. Mitophagy, a selective autophagy of mitochondria, can efficiently degrade, remove and recycle the malfunctioning or damaged mitochondria, and is crucial for quality control. In past decades, numerous studies have identified a series of factors that regulate mitophagy and are also involved in carcinogenesis, cancer cell migration and death. Therefore, it has become critically important to analyze signal pathways that regulate mitophagy to identify potential therapeutic targets. Here, we review recent progresses in mitochondrial dynamics, the mechanisms of mitophagy regulation, and the implications for understanding carcinogenesis, metastasis, treatment, and drug resistance.

## Introduction

Mitochondria are the energy-producing cellular compartment, also known as the cell’s “power house,” and act as the primary site for aerobic respiration (Giampazolias and Tait, 2016). Mitochondria not only have this core bioenergy function, but they also provide basic materials for tumor anabolism, tumor cell redox and calcium homeostasis control, transcription regulation and cell death control (Chen and Mellman, 2017). The effects of the host immune system on tumor development, progression, and response to therapy are similarly dependent on tumor mitochondrial metabolism (Wallace, 2012; Vyas et al., 2016). Consequently, mitochondria are expected to be an excellent target to exploit for new anticancer drugs (Porporato et al., 2018).

Mitophagy, the specific autophagy of mitochondria, plays a pivotal role in mitochondrial quality control by clearing damaged mitochondria. Severe defects in mitophagy, are associated with complete impairment of mitochondrial functions, oncogenesis and tumor progression in multiple tumors (Guo et al., 2013; Rosenfeldt et al., 2013; Rao et al., 2014). Hence, the study of mitophagy regulatory mechanisms in cancer-related events is of great significance. Mitochondria are highly dynamic structures. The coordinated cycle of mitochondrial division and fusion (“mitochondrial dynamics”; Tilokani et al., 2018) provides fast morphological adaptation of mitochondria and plays an important role in the regulation of the cell cycle, cellular immunity, apoptosis, and mitochondrial mass (Wai and Langer, 2016). Mitochondrial dynamic dysfunction can directly damage cells through inadequate ATP supply or high production of ROS and NOS. This in turn can lead to abnormalities, such as neurodegenerative diseases, cancer and autoimmune diseases (Vásquez-Trincado et al., 2016). Many human diseases are associated with mutations in mitochondrial core mechanical components and flaws in mitochondrial dynamics (Mishra and Chan, 2016; Chan, 2020).

This review covers the latest progress in mitochondrial dynamics and mechanisms of mitophagy regulation in the context of carcinogenesis, metastasis, cancer therapy and drug resistance, and proposes future directions for developing novel cancer therapy strategies focused on mitochondria.

## Mitochondrial Dynamics

Mitochondrial dynamics is the process of mitochondrial fusion and fission, and determines the shape, quality and quantity of mitochondria (Chan, 2012; Pernas and Scorrano, 2016). Mitochondrial dynamics is closely linked to mitochondrial functions, such as cell proliferation, cell metabolism, and cell migration (Chan, 2006) and is tightly regulated by a variety of proteins.

### Mitochondrial Fission and Fusion

The mitochondrial fission process (mitofission) is mainly mediated by Drp1. Drp1 belongs to a class of GTP-binding proteins and can be recruited from cytoplasm to the mitochondrial membrane with the assistance of mitochondrial receptor proteins Fis1, MFF, MID49, and MID51 (Losón et al., 2013). Then Drp1 located on the mitochondrial membrane can form a ring structure that closely surrounds the mitochondrion (Ingerman et al., 2005), and induces a breakage of the mitochondrial membrane requiring hydrolysis of GTP (Ji et al., 2015). The phosphorylation, ubiquitination and sumoylation of Drp1 can regulate mitofission by influencing Drp1 stability and recruitment (Chang and Blackstone, 2010). Phosphorylation of Drp1 on Ser585 catalyzed by CDK 1/Cyclin B promotes mitofission in mitotic cells (Taguchi et al., 2007), while phosphorylation of Drp1 on Ser637 can inhibit mitofission.

The mitochondrial fusion process (mitofusion) can be divided into outer mitochondrial membrane (OMM) fusion mediated by mitofusin 1 (Mfn1) and mitofusin 2 (Mfn2) and inner mitochondrial membrane (IMM) fusion mediated by OPA1 (Ni et al., 2015). Mfn and OPA1 are dynamin-related GTPases. Mfn1 and Mfn2 expressed on the OMM can interact to mediate fusion between adjacent OMM (Cao et al., 2017). OPA1 located in the IMM together with Mfn1 mediates the fusion of IMM (Cipolat et al., 2004). At present, the exact mechanism of IMM fusion is not clear, but OPA1 has been proved to be necessary. Fusion-related proteins are regulated by posttranslational modifications that effect their abundance and activity (Senft and Ronai, 2016); Mfn1 and Mfn2 activities can be modified by specific phosphorylations, and ubiquitination of the proteins may lead to their degradation.

### The Roles of Mitochondrial Dynamics in Cancer

Mitochondrial dynamics are closely related to the occurrence and metastasis of tumors. Environmental alterations around a cell lead to mitochondrial dynamics change, which is also a mechanism for cancer adaption. There are disorders of mitochondrial dynamics in many cancers; up-regulation of fission-related proteins and down-regulation of fusion-related proteins is found in many types of cancer (Rehman et al., 2012; Zhao et al., 2013; Huang et al., 2017). For example, there is an increase of mitochondrial fragmentation and phosphorylation activation of Drp1 in brain tumor initiation cells (Xie et al., 2015). Additionally, the expression of Mfn2 in lung cancer is lower than that in normal tissues, and overexpression of Drp1/Mfn2 can affect mitochondrial dynamics and inhibit the proliferation of lung cancer cells (Rehman et al., 2012). The findings imply that Drp1/Mfn2 exerts different effects in different cancers.

Targeting therapy based on mitochondrial dynamic-related factors is becoming a potential method for cancer therapy. For instance, hypoxia increases the expression of Drp1 and mitofission in glioblastoma cells, enhances tumor migration, and treatment with Mdivi-1 (DRP1 inhibitor) reduces hypoxia-induced migration (Wan et al., 2014). Mdivi-1 is the best drug to use to characterize mitochondrial dynamics (Cassidy-Stone et al., 2008). It can induce proliferation arrest and apoptosis of tumor cells (Rehman et al., 2012; Wang et al., 2015), but it has some cytotoxicity to normal cells. The chemosensitivity of cancer cells can be improved by mitofission. By down-regulating the phosphorylation of Drp1 at Ser637, Piperlongumine can induce the apoptosis of cisplatin-resistant ovarian cancer cells (Farrand et al., 2013; Table 1). Moreover, treatment with a lectin, *Sambucus nigra* agglutinin, induces mitofission-mediated apoptosis by stimulating Drp1 translocation (Chowdhury et al., 2017). However, there is a reverse action for Drp1 in cancer treatment. The inhibition of Drp1-mediated mitochondrial fission results in the sensitization of ovarian cancer cells to cisplatin (Yang et al., 2017). Thus, the mechanisms of mitochondrial dynamics can rationally be used as a potential target for cancer therapy.

**TABLE 1:** Compounds targeting mitochondria in the treatment of cancer.

**Compound**	**Cancer type**	**Effect**	**References**
Mdivi-1	Lung cancer	Inhibitor of Drp1 and proapoptotic effects	Rehman et al., 2012
Piperlongumine	Ovarian cancer	Activate Drp1 and induce both fission and apoptosis	Farrand et al., 2013
Lectin	Ovarian cancer	Activate AKT signal pathways and de-phosphorylates Drp1	Chowdhury et al., 2017
DHE	Lung cancer	Induce mitochondrial dysfunction, mitophagy and apoptosis	Chang et al., 2016
Nanomicelle	Non-small cell lung carcinoma	Trigger excessive mitophagy/autophagy and energy depletion	Zhu et al., 2020
CerS1/C18 pyridinium ceramide	Hypopharyngeal cancer	Target autophagosomes to mitochondria and induce lethal mitophagy	Sentelle et al., 2012
AT 101	Glioma	Induce mitochondrial dysfunction and mitophagy	Meyer et al., 2018
Sodium selenite	Glioma	Induce mitochondrial damage and subsequent mitophagy	Kim et al., 2007
LCL-461	Acute myeloid leukemia	Induce lethal mitophagy	Dany et al., 2016
Liensinine	Breast cancer	Sensitize cancer cells to chemotherapy through DNM1L-mediated mitofission	Zhou et al., 2015
Isoliensinine	Breast cancer	Induce cancer cell apoptosis through ROS and p38 MAPK/JNK activation	Zhang et al., 2015
Mito-CP	Colon cancer	Induce changes in mitochondrial bioenergetics, block mTOR-mediated proliferation and induce mitophagy	Boyle et al., 2018
Mito-Metformin			
Abrus agglutinin	Glioblastoma	Trigger ceramide production, induce ER stress and ROS to promote mitophagy	Panda et al., 2018
BAY 87-2243	Melanoma	Inhibit mitochondrial complex I, trigger mitophagy	Basit et al., 2017
B5G1	Liver cancer	Inhibit PINK1-Parkin dependent mitophagy	Yao et al., 2019

## Molecular Mechanisms of Mitophagy Regulation

Mitophagy can selectively remove damaged or redundant mitochondria. Mitophagy is an essential part of mitochondrial stress response and homeostasis regulation, also playing a regulatory role in mitochondrial quality control (Abeliovich et al., 2013). Thus, when the mechanism of mitophagy is impaired, mitochondrial function is reduced or mitochondrial redundancy is caused, which will affect cell homeostasis and lead to the occurrence of related diseases.

**FIGURE 1:**
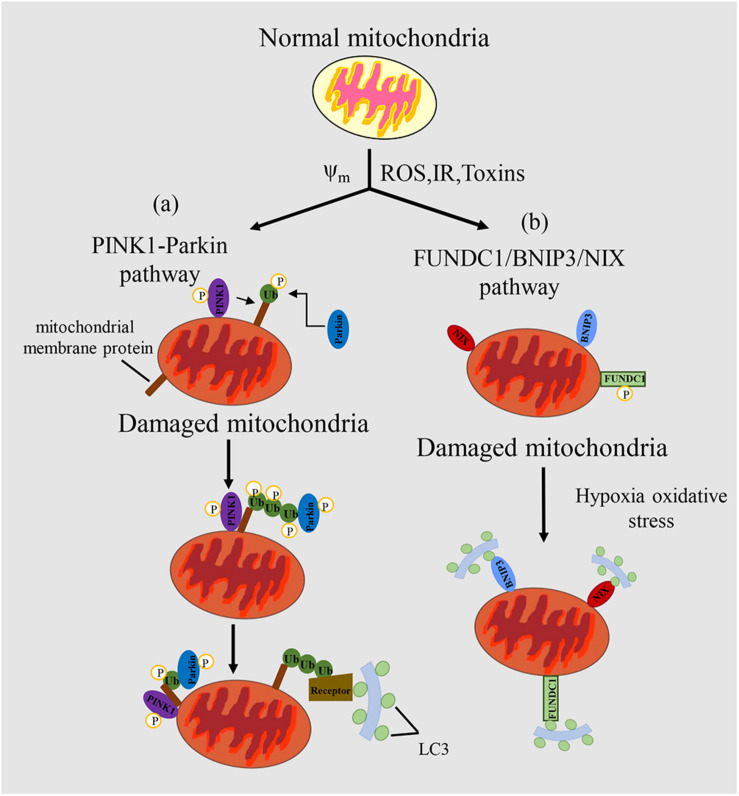
Mechanism of mitophagy regulation in mammals. **(a)** In the PINK/PARKIN pathway, upon mitochondrial impairment or loss of mitochondrial potential, PINK1 can phosphorylate various target proteins, such as ubiquitin. Then, PINK1 recruits Parkin, and Parkin can amplify the signal through ubiquitination of miyochondrial surface receptor proteins. Receptor proteins can recognize the ubiquitinated proteins, which promotes mitochondria to form autophagosomes and eventually degrade them. **(b)** In the FUNDC1/BNIP3/NIX pathway, LC3 can be recognized by receptor proteins of mitochondria to promote the complementation of phagophores, thus targeting the mitochondrion for mitophagy. Mitochondrial outer membrane proteins FUNDC1, BNIP3 or NIX bind to LC3-II through their cytoplasmic LIR motifs to promote selective clearance of mitochondria. Δψ_*m*_, mitochondrial potential; ROS, reactive oxygen species; IR, ischemia/reperfusion; Toxins, poisons produced naturally by organisms.

Principally, mitophagy pathway can be divided into two types: ubiquitin-mediated mitophagy and receptor-mediated mitophagy. The well-studied pathways are PINK1-Parkin mediated ubiquitin pathway and FUNDC1 receptor-mediated pathway, respectively (Figure 1).

### Mechanisms of the Ubiquitin Type Mitophagy Pathway

The PINK1-Parkin mediated regulation of mitophagy is characterized by the ubiquitylation of some key mitochondrial proteins. Parkin, as an E3 ubiquitin ligase, and PINK1, as a serine/threonine kinase, were initially found to be closely related to Parkinson’s disease (Kitada et al., 1998). In normal cells, the PINK1 protein synthesized in the cytoplasm is firstly translocated into the mitochondrial inner membrane, in which is cleaved by PARL protease and degraded by the ubiquitin mediated proteasome pathway (Sekine and Youle, 2018). However, upon mitochondrial impairment or depolarization of mitochondrial membrane potential, the lysis of PINK1 is inhibited, which causes the selective accumulation of PINK1 in OMM (Kondapalli et al., 2012). PINK1 can be activated by autophosphorylation and then phosphorylate Ub at Ser65. Phosphorylated Ub can bind and recruit Parkin from cytoplasm to OMM where PINK1 phosphorylates Parkin at Ser65 and activates its ubiquitin ligase activity (Schubert et al., 2017). Parkin can ubiquitylate a few outer mitochondrial membrane proteins, such as Mfn1, Mfn2, VDAC, MIRO1 (Chan et al., 2011). Ubiquitylated protein such as VDAC can be recognized by LC3 junction proteins in the cytoplasm, including SQSTM1/P62, OPTN, NDP52, TAX1BP1, and NBR1, can bind with this ubiquitinated protein and induce mitophagy by recognizing and binding to LC3 on the autophagy membrane (Lazarou et al., 2015). During this process, Ambra1 is thought to be an interaction protein of Parkin, and its main function is to stimulate cellular autophagy and mitochondrial clearance by motivating the formation of new phagophore (Fimia et al., 2007). Thus, Ambra1 plays the crucial role in cell autophagy and mitophagy.

### Mechanisms of the Receptor-Mediated Pathway

The main feature of the mitophagy pathway is that it is receptor-mediated, via not only protein receptors, but also some lipid molecules (Bingol et al., 2014). ATG32 (Kanki et al., 2009; Okamoto et al., 2009) in yeast and NIX (Novak et al., 2010), BNIP3 (Zhu et al., 2013), FUNDC1 (Liu et al., 2012), FKBP8 (Bhujabal et al., 2017), Bcl2L13 (Murakawa et al., 2015; Otsu et al., 2015), Ambra1 (Strappazzon et al., 2015), PHB2 (Wei et al., 2017), and NLRX1 (Zhang et al., 2019) in mammalian systems have been identified. The common characteristic of these receptor proteins is that they contain a conserved LC3-interacting receptor (LIR) domain, a key domain that binds directly to Atg8/LC3 and other proteins in the family.

Under the starvation conditions, mitophagy occurs in yeast cells. The occurrence may be related to the accumulation of mitochondrial oxidative damage (Farré et al., 2009). Yeast mitophagy is mainly mediated by Atg32, the first receptor that was found to mediate mitophagy (Kanki et al., 2009; Okamoto et al., 2009). Atg32 is a single transmembrane protein with the N-terminal and C-terminal exposed to cytoplasm and mitochondrial stroma respectively (Kondo-Okamoto et al., 2012). After mitochondrial damage, Protein kinase 2 can promote the phosphorylation of Atg32 in Ser114 and Ser119. Phosphorylated Atg32 forms a complex with Atg11, which is the first step of mitochondrial degradation (Hirota et al., 2012) and essential for the initiation of mitophagy in a pre-autophagosomal structure (PAS). Then Atg11 interacts with Atg8 which enables mitochondrial recognition by autophagy elements and recruitment into autophagy precursors. Atg32 can also directly interact with Atg8, to promote the engulfment of the mitochondria by the phagocytosis membrane. The interaction of Atg32 with Atg8 and Atg11 promotes the formation a mitophagy-initiating polymeric body (Kanki et al., 2011). Under nitrogen starvation conditions, the effect of Atg32-Atg11 is enhanced (Kanki et al., 2011). The knockout of Atg32 does not affect the non-selective autophagy, the cytosol-to-vacuole targeting (Cvt) pathway or the occurrence of peroxidase autophagy, but completely inhibits mitophagy. Atg11 is a binding protein in selective mitophagy that recognizes localized receptor proteins on autophagosomes. Atg8 is implicated in the expansion of phagocytic bubbles. LC3 is the Atg8 homologue in mammals, and is involved in the identification process between autophagosome membrane and target (Kabeya et al., 2000). When Atg32 is absent, cells grow normally and intracellular ROS levels remain unchanged when using non-fermentable carbon as a carbon source (Ashrafi and Schwarz, 2013), suggesting that there is another mitophagy pathway independent of Atg32.

BNIP3 and BNIP3L/NIX are both OMM proteins that can trigger mitophagy under hypoxia through binding of their LIR sequences with LC3. NIX-mediated mitophagy was firstly found in the maturation process of red blood cells (Sandoval et al., 2008). The dimerization of BNIP3 is necessary for its interaction with LC3 that is promoted by the phosphorylation of two serine sites (Ser17 and Ser24) in BNIP3 promotes the interaction with LC3. BNIP3 and Nix have different tissue specificity. Nix is mainly expressed in hematopoietic tissue (Diwan et al., 2007), while BNIP3 is widely distributed in heart, liver and muscle. BNIP3 is regulated by RB, NF-κβ, FOX03, Ras and p53, while p53 can also regulate Nix (Mammucari et al., 2007). Hypoxia activates hypoxia inducible factor 1α (HIF1α), which can increases the expression of BNIP3 (Subarsky and Hill, 2003). Additionally, BNIP3 knockout can up-regulate the expression of NIX, but this upregulation cannot make up for the decrease of mitochondrial autophagy caused by BNIP3 knockout (Shi et al., 2014). Hypoxia-induced mitophagy can also be mediated by the outer mitochondrial membrane (OMM) protein termed FUN14 domain-containing protein 1 (FUNDC1) (Liu et al., 2012). FUNDC1 induces mitophagy in a manner independent of the Parkinson’s disease-associated E3 ubiquitin ligase Parkin and does so through the direct binding of its LIR motif to LC3. Phosphorylation or ubiquitin modification of FUNDC1 can affect mitophagy. PGAM5 and ULK1 catalyze de-phosphorylation and phosphorylation of FUNDC1 on Ser13 and Ser17 respectively, which transduces hypoxia signals and induces FUNDC1-mediated mitophagy (Chen et al., 2014). In addition, MARCH5 promotes the degradation of FUNDC1 by ubiquitin modification, and ultimately inhibits the occurrence of mitophagy (Chen Z. et al., 2017).

## The Roles of Mitophagy in Cancer

**FIGURE 2:**
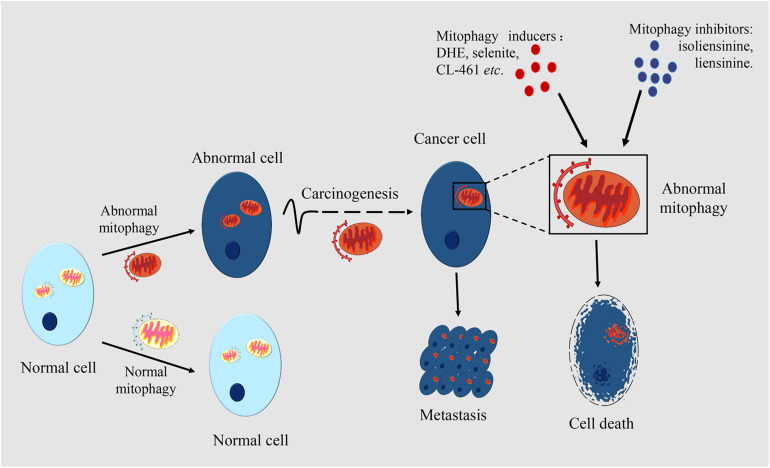
The roles of mitophagy in cancer. Mitophagy occurs in many types of cell. Mitophagic levels are usually enhanced or inhibited in cancer cells, which is different from normal cells. Mitophagy is associated with the occurrence and metastasis of cancers. In certain periods of tumorigenesis, limited mitophagy promotes the development of the tumor, while in established tumors, mitophagy can help the survival of tumor cells. Mitophagy also plays an important role in cancer metastasis. By making mitophagy as the target, inducers or inhibitors of mitophagy can have an anticancer effect through regulating the level of mitophagy. Damaged mitochondria in cancer cells are rapidly cleared through mitophagy which mediates the drug resistance of cancer cells.

Mitochondria play important roles in cellular metabolism. Mitophagy maintains mitochondrial homeostasis and normal physiological function, while abnormal mitophagy will cause multiple diseases, such as heart disease, neurodegenerative disease and muscle disease. However, the role of mitophagy in tumor development and progression needs further study. More and more studies have found that there are different levels of mitophagy in diverse cancers including liver cancer, rectal cancer, breast cancer, lung cancer, which reflects the close relationship between mitophagy and cancer (Chourasia et al., 2015a). The changes in the complex functions of mitochondria have important impacts on the growth and progression of cancer (Panigrahi et al., 2019). The roles of mitophagy in cancer are shown in Figure 2.

### Mitophagy and Carcinogenesis

Dysfunction in mitophagy has a close connection with tumorigenesis and tumor development (Chang et al., 2017). The role of mitophagy differs in different stages of tumor development. In the early stage of tumorigenesis, mitophagy maintains normal cell metabolism and inhibits tumorigenesis, while in the later stage of tumor development, the occurrence of mitophagy improves cell tolerance and promotes the development of the tumor.

The loss, mutation or functional change in diverse pivotal genes results in the accumulation of damaged mitochondria and suppression of mitophagy, which eventually stimulates tumorigenesis (Panigrahi et al., 2019). PINK1/Parkin pathway is known as the key pathway of mitophagy (Youle and Narendra, 2011). The loss of function of Parkin can inhibit mitophagy and promote carcinogenesis in various cancer models. The inhibition of mitophagy further results in an upsurge of reactive oxygen species which influence on the function of cells, tissues, organs and even whole systems. An early study found that mice with Parkin knock-out are more susceptible to spontaneous development of hepatocellular carcinoma (HCC) than the mock mice (Fujiwara et al., 2008). Moreover, the result also showed that Parkin-deficient hepatocytes continue to proliferate and exert an antiapoptotic effect by upregulating endogenous follistatin, which eventually promotes cancer progress.

PARK2 gene which is located on human chromosome 6 (FRA6E, 6q26), encodes Parkin protein and is very sensitive to gene mutation (Burrell et al., 2013). Parkin mutations are often detected in various tumors such as lung cancer (D’Amico et al., 2015), glioma (Maugeri et al., 2015), and colon cancer (Poulogiannis et al., 2010). The appearance of mutated forms of PARK2 gene is distinct in different cancers (Bernardini et al., 2017). For example, PARK2 amplification is common in sarcomas and uterine cancers, but damaging mutations of PARK2 such as deletion or loss of function often occur in other tumors. It was reported that up-regulation of Parkin expression can inhibit cancer cell growth in hepatocarcinoma, colon cancer, breast cancer and glioblastoma (Wang et al., 2004; Poulogiannis et al., 2010; Tay et al., 2010; Veeriah et al., 2010), while down-regulation of Parkin expression promotes cell proliferation and tumorigenesis in pancreatic cancer (Sun et al., 2013). When PARK2 knock-out mice were hybridized with colorectal cancer adenomatous polyposis mice, the development of intestinal adenoma in neonatal mice accelerated rapidly, and the diversity of polyps increased (Poulogiannis et al., 2010), indicating that PARK2 is a tumor suppressor gene.

Mitophagy has gradually become recognized as a critical adaptation in response to hypoxia. Just as cells can reduce the number of mitochondria and not only limit the production of reactive oxygen clusters, they can also maximize the use of available oxygen. Under hypoxic condition, BNIP3 and its ligand Nix are two main receptors in mitophagy. Like Parkin, Nix also can play an important role in tumorigenesis. Under hypoxia conditions, HIF-1 induces up-regulation of Nix and BNIP3 expression in tumor tissues (Sowter et al., 2001). Nix is highly expressed in hypoxic tumor cells, and Nix-mediated mitophagy promotes glioblastoma survival. The silencing of Nix impairs the clearance of ROS, cancer stem cell maintenance, and inhibits the survival of tumor cells (Jung et al., 2019). Oncogenic KRAS induces NIX-mediated mitophagy, and promotes pancreatic carcinogenesis, but the loss of NIX leads to the restoration of mitochondrial function, which significantly delays pancreatic cancer progression (Humpton et al., 2019). However, another report showed that up-regulated expression of NIX induced by p53 under hypoxia promotes tumor cells apoptosis, while knockout of Nix gene accelerates tumor growth (Fei et al., 2004).

Another mitophagy-related protein FUNDC1 also plays a vital role in carcinogenesis. In early-stage cervical cancer patients, it was found that FUNDC1 has a higher expression level than adjacent normal cells (Hou et al., 2017). Moreover, the deletion of FUNDC1 in tumor cells obviously inhibited cell proliferation, triggered cell apoptosis and increased the sensibility of tumor cell to chemo-radiotherapy such as cisplatin and ionizing radiation. FUNDC1 accumulates in most human HCC. In hepatocytes, FUNDC1 knock-out promotes the initiation and development of diethylnitrosamine (DEN)-induced primary HCC; in contrast when FUNDC1 is transferred into hepatocytes it inhibits hepatocarcinogenesis (Li et al., 2019). In this context, specific deletion of FUNDC1 in hepatocytes leads to the accumulation of dysfunctional mitochondria, and further stimulates a series of events including the activation of inflammasome and JAK/STAT signaling. This suggests that mitophagy can inhibit the occurrence and development of HCC by inhibiting the activation of inflammatory bodies.

### Mitophagy and Cancer Metastasis

During cancer progression, some cancer cells convert the aerobic respiration of mitochondria into glycolysis to maintain cancer cell’s energy needs. This is called the Warburg effect (Bensinger and Christofk, 2012). Cancer cells are typically characterized by a Warburg effect, abnormal quality control of mitochondria, production of ROS, regulation of cell redox state, and lack of apoptosis signals (Panigrahi et al., 2019). By eliminating dysfunctional mitochondria, mitophagy can promote tumor cell survival as an adaptation to stress. In the process of mitophagy, Parkin increases the level of oxidative metabolism and inhibits the Warburg effect downstream of the p53 tumor suppressor, which is likely to be achieved by enhancing the integrity of mitochondrion (Zhang et al., 2011). Thus, as a p53 target gene, Parkin may exhibit tumor suppressive activity. Another study further confirmed that in breast cancer, Parkin inhibited tumor migration and invasion through targeting HIF-1α for ubiquitination and degradation (Deng et al., 2017). When the Parkin gene was transferred into breast cancer cells with a deleted Parkin gene the progression of breast cancer and the rate of metastasis were significantly decreased. Notably, there is also emerging evidence that Parkin may promote cancer metastasis. Compared with normal dermatic tissues, pathological analysis showed that the expression of Parkin and cancer metastasis was obviously enhanced in melanoma (Lee et al., 2018). Further results indicated that the loss of Parkin inhibited tumor formation and metastasis of melanoma by suppressing Mfn2 ubiquitination (Lee et al., 2018). The cause of the opposite result for Parkin’s role in cancer metastasis could be due to different mechanisms for metastasis in several tumor types.

BNIP3 as a pro-apoptotic protein, can inhibit the fusion of damaged mitochondria and enhance mitophagy (Gustafsson, 2011). However, the role of BNIP3 in cancer metastasis is varied in different cancers. It is frequently found that the high expression of BNIP3 that occurs in many kinds of malignancies, such as salivary gland adenoid cystic carcinoma (Chen et al., 2015), endometrial cancer (Giatromanolaki et al., 2008), DCIS of breast and cervical cancer (Leo et al., 2006) is associated with the aggressiveness of the tumor and poor prognosis. The loss of BNIP3 makes mitophagy proceed abnormally and increases mitochondrial ROS levels, which is related with tumor metastasis in TNBC (Chourasia et al., 2015b). In DCIS, the expression of BNIP3 and Nix are increased, while BNIP3 is not expressed in invasive carcinoma, which is related to tumor cell proliferation index and lymph node metastasis (Sowter et al., 2003). It is also suggested that the higher expression of BNIP3 mediates excessive mitophagy, which inhibits HCC metastasis (Shi et al., 2018). As a small GTPase, Rheb can not only interact with BNIP3 and NIX, but also effectively acts on the upstream region of mTOR pathway to promote cell growth (Li et al., 2007). The inhibition of BNIP3 by Rheb under the interaction of Rheb and BNIP3 decreases the activity of mTOR signal, and suppresses cell growth, which corresponds to the role of BNIP3 in tumor inhibition (Ray et al., 2000).

However, similarly to PINK1/PARKIN, BNIP3 can not only serve as a tumor suppressor, but can also exert oncogenic activity. The epigenetic silencing of BNIP3 could enhance the aggressiveness and metastasis of pancreatic cancer cells, and contribute to resistance to hypoxia-induced cell death in pancreatic cancer (Okami et al., 2004). Besides, up-regulation of BNIP3 has also been reported to accelerate cancer cell migration and invasion (Maes et al., 2014; Wu H. et al., 2015). The dual roles of BNIP3 in cancer progress and metastasis could be due to different interact with different factors through its Bcl-2 homology 3 (BH3) domain, also to the heterogeneity of different tumors.

### Mitophagy and Cancer Therapy

It is clear that mitophagy is beneficial in maintaining normal healthy physiological processes. Mitophagy shares common regulatory pathways with carcinogenesis and cancer cell death which converge at mitochondria (Lu et al., 2013). Thus, it provides therapeutic targets for removing cancer cells and inducing cancer cell apoptosis; therapies can aim at the crosstalk signals between the processes of mitophagy and cancer progression. Concurrently, mitochondria have a function on cell apoptosis and the ability to inhibit apoptosis and resist cell death is one of the well-established hallmarks of cancer (Hanahan and Weinberg, 2011), which implies the potential for mitochondria-mediated apoptosis in cancer therapy.

In a similar way to macroautophagy, mitophagy also plays dual roles in cancer therapy, including the induction of cell death and promotion of cell survival (Panigrahi et al., 2019). Generally, chemotherapy induces mitochondrial dysfunction and oxidative stress to generate the cytotoxic effects on cancer cells which result in the generation of mitophagy (Cao et al., 2019). Excessive mitophagy causes the loss of functional mitochondria, and further destruction of cellular energy requirement, leading to a form of cancer cell death called mitophagic cell death. However, another role of mitophagy in cancer is that of improving the cell internal environment by clearing abnormal mitochondria, which can result in better fitness in the aggressive environments (Araki et al., 2019).

Some compounds that activate mitophagy are gradually being identified as chemotherapeutic drugs. Dihydroergotamine tartrate (DHE) is a common drug for migraine. A study has reported that DHE induces lung cancer cell death by mitophagy and mitochondria-dependent cell apoptosis (Chang et al., 2016). Mechanistically, DHE could induce PINK1/PARKIN activation, ROS production and cell apoptosis, and reduce membrane permeability and destroy ATP synthesis, finally activating mitophagic cell death. This suggests that DHE might be a promising anticancer medicine for lung cancer. More recently, Zhu et al. (2020) reported a novel anticancer strategy based on excessive mitophagy. They used a novel nanomicelle that targets mitochondria to selectively damage mitochondria in tumor cells, thereby further activating excessive mitophagy pathway-driven lethal energy depletion and phototherapy. An *in vivo* study showed that CerS1/C18 pyridinium ceramide efficiently induces lethal mitophagy and abrogates growth of tumor xenograft in various solid cancers (Sentelle et al., 2012). In glioma cells, a novel BH3-mimetic AT101 (Meyer et al., 2018) and sodium selenite (Kim et al., 2007) induced an excessive mitophagic cell death that suggests lethal mitophagy as the tumor-suppressor mechanism. Similarly, LCL-461, a mitochondria-targeted ceramide analog, could also kill crenolanib-resistant acute myeloid leukemia (AML) cells through lethal mitophagy (Dany et al., 2016). However, other studies revealed that some compounds that inhibit mitophagy exert antitumor effects in breast cancer. Liensinine, a new inhibitor of autophagy and/or mitophagy, could enhance the sensitivity of breast cancer cells to chemotherapy via mitochondrial fission mediated by DNM1L (Zhou et al., 2015). Isoliensinine could induce cell apoptosis in TNBC by the production of ROS and the activation of p38 MAPK/JNK and improve anti-breast cancer effects by inhibiting mitophagy (Zhang et al., 2015).

In addition to the use of mitophagy-associated compounds, the most prominent approach for mitophagy-mediated cell death is by targeting regulation of mitophagy associated proteins by a selective reduction in mitochondrial compartments (Zhou et al., 2015; Panigrahi et al., 2019). Several mitophagy-related proteins PINK1, Parkin, BNIP3, CDKN2A, PUMA, ULK1, and TR3 could induce mitochondrial damage and activate mitophagy, and ultimately lead to cell death. Activated TR3 receptor could result in irreversible mitophagic cell death and excessive clearance of mitochondria in the melanoma cells to TR3-specific compounds (Wang et al., 2014). Mitochondria-targeting drugs (Mito-CP and Mito-Metformin) trigger mitophagy and inhibit the proliferation of colon cancer cell through an AMPK-mTOR-ULK1-dependent pathway (Boyle et al., 2018). Moreover, lethal mitophagy also happens through activation of ceramide stress in human cancer cells (Sentelle et al., 2012), and PUMA-mediated mitophagy by Abrus agglutinin promotes cell apoptosis by the generation of C18 ceramide or the overexpression of CerS1 (Panda et al., 2018).

Inhibition of the tumor promotion role of mitophagy is another promising strategy for cancer therapy. Many studies reported that mitophagy-associated molecules PINK1, Parkin, BNIP3, FUNDC1 promote tumor development, and provide targets for therapeutic inhibition in multiple cancers (Tan et al., 2007; D’Amico et al., 2015; Liu et al., 2018; Hui et al., 2019). For example, the silencing of PINK1 can not only inhibit the proliferation and migration of lung cancer cells, but can also induce cell apoptosis (Liu et al., 2018). Furthermore, high expression of PINK1 in ESCC patients receiving neoadjuvant therapy is related to low chemotherapy effects and poor prognosis (Yamashita et al., 2017). PINK1-mediated excessive mitophagy promotes survival of cancer cells resistant to chemotherapy, implying that suppression of mitophagy can restore the chemo-sensitivity of ESCC cells (Yamashita et al., 2017). Moreover, overexpression of FUNDC1 promotes growth of cervical cancer cells, and inhibition of expression enhances the sensitivity to cisplatin treatment and ionizing radiation (Hou et al., 2017). This shows that the regulation of mitophagy-associated molecules could affect the outcome of cancer therapy.

### Mitophagy and Drug Resistance

Tumor resistance raises complex questions involving multiple steps and factors. Recent study has shown that both the efficacy of tumor chemotherapy and the degree of drug resistance are influenced by mitophagy, probably because cancer cells rapidly sweep away damaged mitochondria through mitophagy to mediate their own drug resistance (Yamashita et al., 2017). Consistent with this, the toxicity of most chemotherapeutics is at least partly due to the induction of mitochondrial dysfunction and oxidative stress (Hockenbery, 2010).

The common chemotherapeutic drugs such as cisplatin, paclitaxel, doxorubicin (Dox) and 5-fluorouracil (5-FU) can kill cancer cells in early treatment of multiple solid cancers. However, the occurrence of drug resistance often leads to treatment failure due to autophagy or mitophagy (Oun et al., 2018). For instance, activation of autophagy triggered by overexpression of galectin-1 leads to chemotherapy resistance of epithelial ovarian cancer to cisplatin (Chen L. et al., 2017) and a similar result has been observed in liver cancer (Su et al., 2016). In addition, hypoxia-induced mitophagy accounts for lung cancer cells resisting cisplatin in an HIF1α- and HIF2α-dependent manner (Wu H. et al., 2015). Notably, an E3 ubiquitin ligase ARIH1 highly expressed in cancer cells could trigger mitophagy in a PINK1-dependent and Parkin-defective way; this countered the chemotherapy-induced cell death in breast cancer and lung adenocarcinomas, eventually resulting in cancer cell resistance (Villa et al., 2017).

However, the desired anticancer results can be achieved through regulation of autophagy or mitophagy. On one hand, suppression of autophagy by *Atg* gene knockdown could activate the BNIP3-mediated cancer cell death pathway after treatment with cisplatin (Wu H. et al., 2015). Similarly, enhanced sensitivity of multidrug-resistant cancer cells to betulinic acid analog B5G1 that was induced by the inhibition of PINK1-Parkin dependent mitophagy resulted in liver cancer cell death (Yao et al., 2019). On the other hand, inducement of mitophagy could improve drug sensitivity of cancer cells. A recent study found that mitophagy-dependent necrosis and ferroptosis can be induced through the suppression of mitochondrial respiratory chain by BAY 87-2243 in melanoma cells (Basit et al., 2017). In addition, through DNM1L-mediated mitochondrial fusion, mitophagy can enhance the sensitivity of breast cancer cells to liensinine (Zhou et al., 2015). These results implicate mitophagy in drug resistance call for further exploration in different cancers.

CSCs are usually resistant to chemotherapy. Interestingly, mitophagy participates in CSCs-mediated drug resistance. In OSCC, the CD44^+^/ABCB1^+^/ADAM17^+^cells behave with the characteristics of CSCs and exhibit chemo-resistance through the modulation of mitophagy (Naik et al., 2018). BNIP3/NIX-mediated mitophagy contributes to the dox-resistance to CSCs in colorectal cancer and the silencing of BNIP3L both prevents mitophagy and increases the sensitivity to doxorubicin therapy (Yan et al., 2017), which suggests the importance of mitophagy in drug resistance for cancer therapy targeting CSCs.

Furthermore, drug resistance regulated by mitophagy also restricts the efficiency of radiotherapy. A recent study has shown that mitophagy induced by p53/BNIP3 exerts an important effect on the viability of HNSCC cancer cells following radiotherapy by maintaining the integrity of mitochondrial (Chang et al., 2019). BNIP3-dependent mitophagy in the radio-resistant cancer cells relies on wild type p53 status to limit glycolytic remodeling, highlighting the potential use of drugs targeting glycolysis as an alternative strategy for overcoming radio-resistant cancers.

## Conclusion and Perspective

Mitophagy appears to be a pivotal cellular event that disposes of dysfunctional mitochondria by autophagic degradation. Although significant advances have been made in delineating mitophagy regulation and the role of mitophagy in carcinogenesis and cancer therapeutics, it is still an intractable problem to understand how mitophagy acts either as a suppressor or an inducer in cancer therapy. Moreover, the homeostasis and regulation of mitochondrial dynamics and mitophagy retain a complexity that requires more exploration. For example, what is the relationship of mitochondrial dysfunction to the maintenance of CSCs self-renewal in tumor progression? What are the molecular crosstalks and interactions between mitophagy and key oncogenic signaling pathways? Can the regulation of mitophagy can be beneficial to molecular targeting in cancer therapy, for example with checkpoint inhibitors (anti-PD-1, PD-L1, and CTLA4), oncolytic virotherapy, molecular immunotherapy? How might we adjust cancer cell mitophagy behavior in relation to specific oncometabolites in various cellular stresses, including hypoxia, nutrient deficiency, and elevated ROS level? How might we best exploit the targeting therapy strategies based on mitochondria and mitophagy by enhancing drug uptake into cancer cells? How does mitophagy further orchestrate the crosstalk that constitutes the metabolic regulation system of cancer cells, which is the key step for maintaining the balance between glycolysis and OXPHOS? In consideration of such questions, further studies will undoubtedly focus on the exploration of the molecular mechanisms of mitophagy in cancer events, the identification of novel mitophagic modulators and the development of promising mitophagy-based cancer treatments.

## Author Contributions

YW, Y-TC, H-HL, and L-LZ performed the literature searches and wrote the manuscript. FH received a grant for this project, conceived the idea, and reviewed the drafts. YW contributed to the conceptual idea and supervised the writing process. CY gave suggestions and significantly refined the manuscript.

## Conflict of Interest

The authors declare that the research was conducted in the absence of any commercial or financial relationships that could be construed as a potential conflict of interest.

## Abbreviations

ABCB1: *P*-glycoprotein
ADAM17: A disintegrin and metalloproteinase
Ambra1: Autophagy and Beclin 1 Regulator 1
ARIH1: Ariadne RBR E3 Ubiquitin Protein Ligase 1
AT 101: (-)-gossypol
Atg: autophagy-related protein
ATP: adenosine triphosphate
Bcl2L13: BCL2 Like 13
BNIP3: BCL2/adenovirus E1B 19 kDa protein-interacting protein 3
BNIP3L/NIX: BNIP3-like
CDK 1: cyclin dependent kinase 1
CDKN2A: cyclin-dependent kinase inhibitor 2A
CSC: cancer stem cell
CTLA4: cytotoxic T lymphocyte-associated antigen-4
Cvt: cytoplasm to vacuole targeting
DCIS: ductal carcinoma *in situ;*
DNM1L: dynamin-1-like protein
Drp1: dynamin-related protein 1
ESCC: esophageal squamous cell carcinoma
Fis1, fission: mitochondrial 1
FKBP8: FK506-binding protein 8
FOX03: Forkhead Box O3
FUNDC1: FUN14 domain-containing protein 1
GTP: guanosine triphosphate
GTPases: guanosine triphosphatase
HCT8: Human Colon Tumor (HCT8) Cell Line
HNSCC: head and neck squamous cell carcinoma
JNK: c-JunN-terminal kinase
KRAS: Kirsten Rat Sarcoma Viral Oncogene
LC3: microtubule-associated proteins 1A/1B light chain 3A
LIR: LC3-interacting domain
MAPK: mitogen-activated protein kinase
MARCH5: membrane-associated RING finger protein 5
Mdivi-1: mitochondrial division inhibitor 1
MID49: mitochondrial dynamics protein of 49 kDa
MID51: mitochondrial dynamics protein of 51 kDa
MIRO1: Mitochondrial Rho GTPase 1
Mito-CP: 3-carboxyl proxyl (CP) nitroxide
mTOR: mammalian target of rapamycin
NBR1: neighbor of BRCA1 gene protein
NDP52: nuclear dot protein 52
NF- κβ: nuclear factor kappa beta
NLRX1: NLR (Nucleotide-binding domain and leucine-rich repeat-containing receptors) family member X1
NOS: nitric oxide synthase
OPA1: optic atrophy 1
OPTN: optineurin
OSCC: oral squamous cell carcinoma
OXPHOS: oxidative phosphorylation
PARK2: gene encoding Parkin
PARL: presenilin-associated rhomboid-like
PD-L1: Programmed Cell Death 1 Ligand 1
PGAM5: hosphoglycerate mutase-5
PHB2: Prohibitin 2
PINK1: mitochondrial PTEN-induced kinase 1
PUMA: p53 upregulated modulator of apoptosis
Ras, Rat Sarcoma protein: Small GTP Binding Protein
RB: retinoblastoma
Rheb: Ras homolog enriched in brain protein
ROS: reactive oxygen species
TAX1BP1: TAX1 binding protein 1
TNBC: triple negative breast cancer
TR3: orphan nuclear receptor
ULK1: Unc-51 Like Autophagy Activating Kinase 1
VDAC: Voltage Dependent Anion Channel 1
Yme1L: YME1-Like Protein 1.
